# Assessment of quarter billion primary care prescriptions from a nationwide antimicrobial stewardship program

**DOI:** 10.1038/s41598-021-94308-z

**Published:** 2021-07-16

**Authors:** Mehmet Gönen, Mesil Aksoy, Fatma İşli, Umut Emre Gürpınar, Pınar Göbel, Hakkı Gürsöz, Önder Ergönül

**Affiliations:** 1 Department of Industrial Engineering, College of Engineering, Koç University, İstanbul, Turkey; 2 School of Medicine, Koç University, İstanbul, Turkey; 3 Turkish Medicines and Medical Devices Agency (TMMDA) of Ministry of Health, Ankara, Turkey; 4 Department of Infectious Diseases and Clinical Microbiology, School of Medicine, Koç University, İstanbul, Turkey

**Keywords:** Epidemiology, Bacterial infection

## Abstract

We described the significance of systematic monitoring nationwide antimicrobial stewardship programs (ASPs) in primary care. All the prescriptions given by family physicians were recorded in Prescription Information System established by the Turkish Medicines and Medical Devices Agency of Ministry of Health. We calculated, for each prescription, “antibiotics amount” as number of boxes times number of items per box for medicines that belong to antiinfectives for systemic use (i.e., J01 block in the Anatomical Therapeutic Chemical Classification System). We compared the antibiotics amount before (2015) and after (2016) the extensive training programs for the family physicians. We included 266,389,209 prescriptions from state-operated family healthcare units (FHUs) between January 1, 2015 and December 31, 2016. These prescriptions were given by 26,313 individual family physicians in 22,518 FHUs for 50,713,181 individual patients. At least one antimicrobial was given in 37,024,232 (28.31%) prescriptions in 2015 and 36,154,684 (26.66%) prescriptions in 2016. The most common diagnosis was “acute upper respiratory infections (AURI)” (i.e., J00-J06 block in the 10th revision of the International Statistical Classification of Diseases and Related Health Problems) with 28.05%. The average antibiotics amount over prescriptions with AURI decreased in 79 out of 81 provinces, and overall rate of decrease in average antibiotics amount was 8.33%, where 28 and 53 provinces experienced decreases (range is between 28.63% and −3.05%) above and below this value, respectively. In the most successful province, the highest decrease in average amount of “other beta-lactam antibacterials” per prescription for AURI was 49.63% in January. Computational analyses on a big data set collected from a nationwide healthcare system brought a significant contribution in improving ASPs.

## Introduction

It is time for analysis and interpretation of big data in service of public health. Relevant action plans developed from these analyses have a strong potential to transform the medicine^1^. We here present the benefits of bringing the big data into the field of antimicrobial stewardship programs (ASPs). Antimicrobial resistance as a consequence of irrational antibiotics consumption is one of the greatest public health threats^2^. If the current trend of antimicrobial resistance is not intervened, it is expected that, by 2050, deaths attributed to drug-resistant infections will be around 10 millions annually^3^. Thus, multinational or national efforts for ASPs should be implemented urgently to decrease irrational use and to take antimicrobial resistance under control^4,5^.

Physicians prescribe antibiotics for many patients with acute upper respiratory infections, which are among the most commonly seen acute presentations in primary care^6,7^. At least half of these infections are viral^8,9^, and thus, rationalization of antibiotics use in the treatment of acute upper respiratory infections in primary care is a priority in the prevention of antimicrobial resistance^10,11^. In 2011, Turkey had the highest antibiotics consumption rate among eastern European and non-EU countries, as 42.3 defined daily doses (DDDs) per 1000 inhabitants per day (DID)^12^. Antibiotics are one of the most commonly consumed drug groups in Turkey, and the most commonly used antibiotics were beta-lactams with extended spectrum, macrolides, and fluoroquionolones^13^. In Turkey, nationwide antimicrobial consumption for J01 class antibiotics (β-lactams, tetracyclines, amphenicols, sulphonamides and trimethoprim, macrolides, aminoglycosides, and quinolones) dropped from 42.3 DID in 2011 to 40.4 DID in 2014 and to 35.25 DID in 2017^14^.

The latest report of the Organisation for Economic Co-operation and Development (OECD) Health Policy Studies stated that in 2015 the highest rates of antimicrobial resistance (around 35% in Turkey, Korea, and Greece) were seven times higher than the lowest rates among its member countries. Despite a 15-year hospital antibiotic restriction programme, Turkey still was the OECD country with the highest rate of resistance (38.8%). More specifically, Carbapenem resistance exceeded 90% in *Acinetobacter baumannii* and approached 50% among *Klebsiella pneumoniae* isolates^14^.

In this study, we report our experience in implementation of an assessment strategy that can be used for developing nationwide ASPs and its immediate benefits on public health such as decreasing antibiotics consumption all over the country. Our study had two main incentives. First, Turkey had been reported as one of the countries with highest antibiotics consumption^12^. Secondly, because of universal coverage for more than 80 million citizens and more than three million refugees, Turkey has an unprecedented opportunity to collect high-quality big data from its healthcare system^15^. We continuously monitored antibiotics prescriptions in primary care over the country, analysed the collected data, and used the information extracted to improve our nationwide ASP.

## Materials and methods

### Study design

In Turkey, since 2011 all the prescriptions given by family physicians were recorded in Prescription Information System (PIS) established by the Turkish Medicines and Medical Devices Agency (TMMDA) of Ministry of Health. The PIS is a centralized website used to collect data about prescriptions from the physicians, to summarize the collected data, and to provide the physicians with feedback reports about their prescribing behaviour. Instead of writing paper prescriptions, the physicians prepare prescriptions electronically using the PIS, and these prescriptions are seen and processed by pharmacists when the patient visits a pharmacy, which allows Ministry of Health collect data from the field. For each prescription, PIS recorded information about the gender and birth date of the patient and the physician, date of the encounter, diagnosis codes assigned to this encounter in terms of the 10th revision of the International Statistical Classification of Diseases and Related Health Problems (ICD10), location of the healthcare centre (province and district), type of the healthcare centre (e.g., family healthcare unit (FHU), district hospital, etc.), and prescribed medicines with their Anatomical Therapeutic Chemical (ATC) classification system codes, box counts, box contents, and administration routes.

TMMDA converted all information about patients and physicians into a non-identifiable form by masking their identifiable parts before computational analysis and approved the study with the number 32776236-518-E.74955. Koç University Institutional Review Board approved the study with the number 2016.267.IRB1.029. There was no need for informed consent since we do not report any identifying information of patients and physicians. All computational analyses were performed in accordance with relevant guidelines and regulations.

### Intervention

TMMDA of Ministry of Health is responsible for the rational use of medicines (RUM) efforts in Turkey, and a national action plan was launched in 2014 by TMMDA. Decreasing antibiotics consumption was one of the priority areas in this action plan. Towards this aim, the central body of TMMDA established provincial teams for RUM in all provinces of Turkey. Each provincial team provided the family physicians in their province with individual feedback reports over their antibiotics prescriptions. In these reports, their prescriptions with antibiotics were benchmarked against those of the family physicians in the same province and those of the family physicians in the whole nation. TMMDA achieved coordination between the provinces by regular meetings, organized a national symposium during antibiotics awareness week in November 2015, and performed training and education programs by bringing the family physicians and the leading medical experts together. Small group workshops were held for more than 4000 family physicians who prescribed the highest rates of antibiotics for acute upper respiratory infections. Besides the interventions on the physicians, dissemination campaigns were organized using posters and brochures to increase the public awareness on this issue.

### Monitoring software

We developed a monitoring software for evaluation of the prescriptions written in 2015 and 2016 by family physicians in FHUs. This repository was designed using Elasticsearch technology (https://www.elastic.co), which is an open source distributed search engine, to be able to analyse hundreds of millions of prescriptions in the order of seconds. The user interface was developed using R statistical computing language (https://www.r-project.org) and Shiny package (https://shiny.rstudio.com). This software allows the central body of TMMDA to monitor antibiotics prescribing behaviour of family physicians with respect to different parameters (e.g., location, ICD10 codes of diagnoses, ATC codes of prescribed medicines, etc.) before and after the interventions done for a nationwide ASP.

### Data analysis

Using the monitoring software, we calculated, for each prescription, total “antibiotics amount” as number of boxes times number of items per box for medicines that belong to J01 (i.e., antiinfectives for systemic use), A07AA11 (i.e., rifaximin), or A07AX03 (i.e., nifuroxazide) ATC codes, which was previously used in measuring global antibiotics consumption^16^. The average antibiotics amounts per prescription before and after the intervention (i.e., our nationwide ASP) were compared.

## Results

We included 266,389,209 prescriptions in total (130,790,300 in 2015 and 135,598,909 in 2016) from state-operated FHUs between January 1, 2015 and December 31, 2016. These prescriptions were written by 26,313 individual family physicians in 22,518 FHUs for 50,713,181 individual patients. The mean age of the patients over prescriptions was 44.05, and 59.05% of prescriptions were written for female patients. The 19.60% of prescriptions were for patients 15 years old or younger. The mean age of the physicians over prescriptions was 43.99, and 30.71% of prescriptions were written by female physicians.

At least one antimicrobial was given in 73,178,916 out of 266,389,209 prescriptions (27.47%) during the study period. At least one antimicrobial was given in 37,024,232 out of 130,790,300 prescriptions (28.31%) in 2015 and 36,154,684 out of 135,598,909 prescriptions (26.66%) in 2016.

The most common diagnosis chapter was diseases of the respiratory system (i.e., ICD10 chapter J00-J99), which was used in 99,015,347 out of 266,389,209 prescriptions (37.17%), and the most common diagnosis block was “acute upper respiratory infections” (i.e., ICD10 block J00-J06), which was used in 74,722,756 out of 266,389,209 prescriptions (28.05%) (Table 1).

**Table 1:** Number of prescriptions with “diseases of the respiratory system” (i.e., ICD10 chapter J00-J99) diagnosis and corresponding subcategories.

ICD10 code	Category name	Number of prescriptions	Percentage (%)
J00-J99	Diseases of the respiratory system	99,015,347	37.17
J00-J06	Acute upper respiratory infections	74,722,756	28.05
J02	Acute pharyngitis	23,722,866	8.91
J06	Acute upper respiratory infections of multiple and unspecified sites	22,438,508	8.42
J00	Acute nasopharyngitis [common cold]	13,271,430	4.98
J03	Acute tonsillitis	12,582,753	4.72
J01	Acute sinusitis	5,166,587	1.94
J04	Acute laryngitis and tracheitis	411,718	0.15
J05	Acute obstructive laryngitis [croup] and epiglottitis	7674	0.00
J30-J39	Other diseases of upper respiratory tract	12,066,789	4.53
J20-J22	Other acute lower respiratory infections	9,477,599	3.56
J40-J47	Chronic lower respiratory diseases	8,382,648	3.15
J09-J18	Influenza and pneumonia	1,269,343	0.48
J95-J99	Other diseases of the respiratory system	21,457	0.01
J80-J84	Other respiratory diseases principally affecting the interstitium	6404	0.00
J60-J70	Lung diseases due to external agents	2183	0.00
J85-J86	Suppurative and necrotic conditions of lower respiratory tract	1915	0.00
J90-J94	Other diseases of pleura	701	0.00

### Effect of ASP on antibiotics prescribed in all provinces

**Figure 1:**
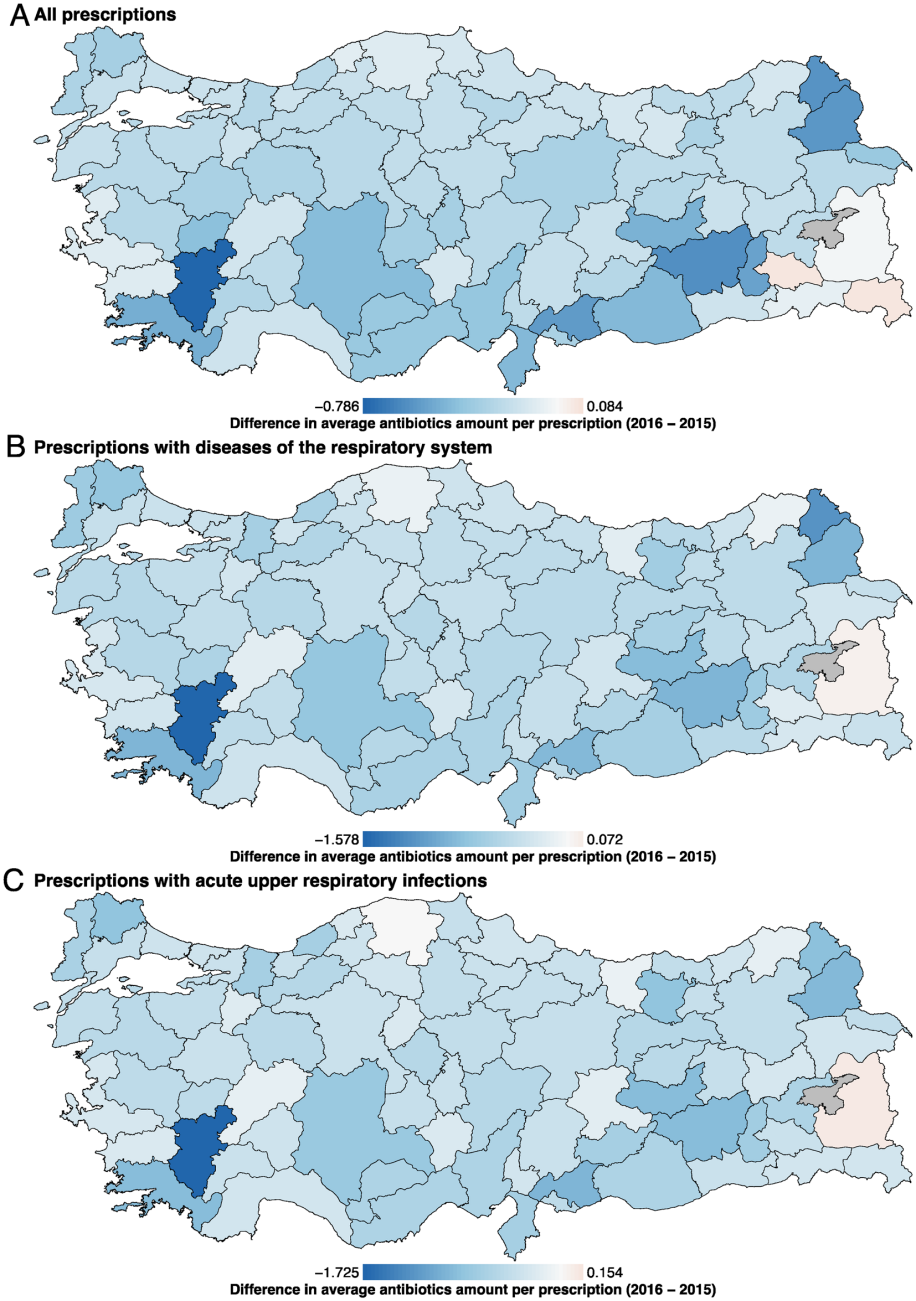
Average antibiotics amount per prescription for each province. (**A**) Over all prescriptions, (**B**) Over prescriptions with “diseases of the respiratory system” (i.e., ICD10 chapter J00-J99), and (**C**) Over prescriptions with “acute upper respiratory infections” (i.e., ICD10 block J00-J06).

The average antibiotics amount per prescription over all prescriptions decreased in 79 out of 81 provinces (Fig. 1A), and overall rate of decrease in average antibiotics amount was 7.94%, where 42 and 39 provinces experienced decreases (range is between 26.03% and −3.48%) above and below this value, respectively (Supplementary Table 1). The highest decrease was observed in Denizli province with 26.03%.

Similarly, average antibiotics amount over prescriptions with “diseases of the respiratory system” diagnosis decreased in 80 out of 81 provinces (Fig. 1B), and overall rate of decrease in average antibiotics amount was 8.51%, where 32 and 49 provinces experienced decreases (range is between 28.03% and −1.49%) above and below this value, respectively (Supplementary Table 2). The highest decrease was again observed in Denizli province with 28.03%.

The average antibiotics amount over prescriptions with “acute upper respiratory infections” diagnosis decreased in 79 out of 81 provinces (Fig. 1C), and overall rate of decrease in average antibiotics amount was 8.33%, where 28 and 53 provinces experienced decreases (range is between 28.63% and −3.05%) above and below this value, respectively (Supplementary Table 3). The highest decrease was once again observed in Denizli province with 28.63%.

### Effect of ASP on antibiotics prescribed for acute upper respiratory infections

**Figure 2:**
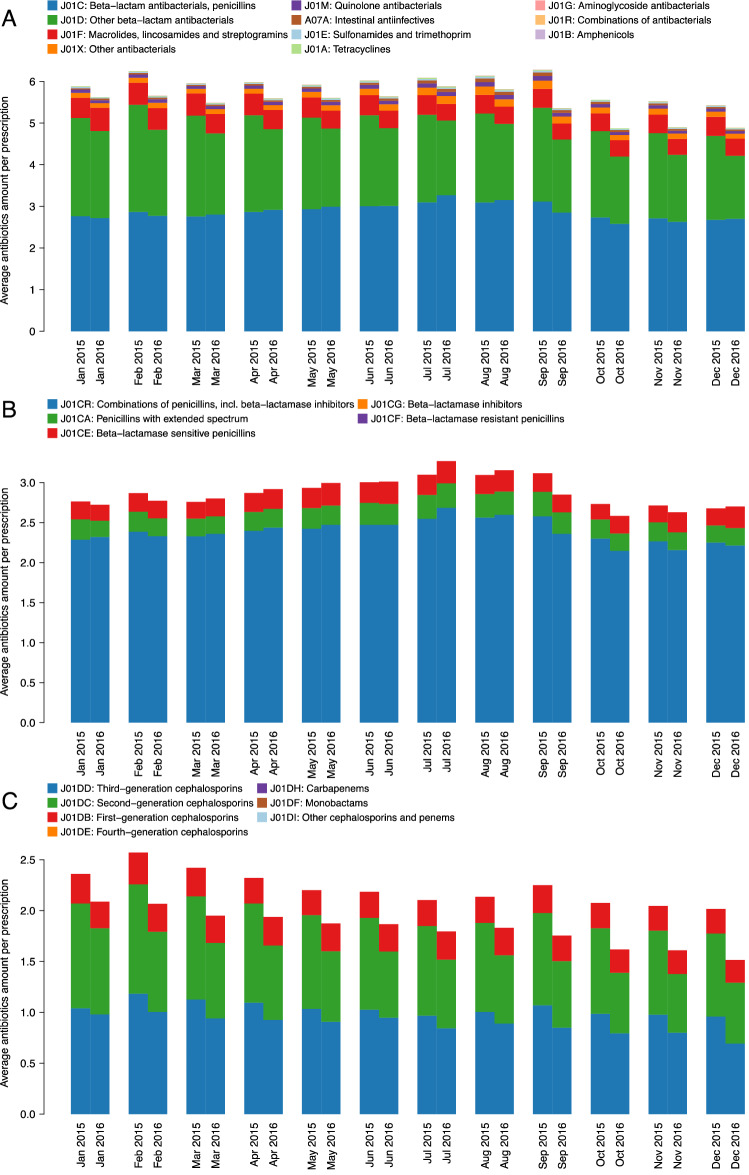
Monthly average antibiotics amounts per prescription for Turkey among the diagnosis of “acute upper respiratory infections” in 2015 and 2016. (**A**) Breakdown with respect to third level ATC codes, (**B**) Breakdown with respect to “beta-lactam antibacterials, penicillins” (i.e., ATC code J01C), and (**C**) Breakdown with respect to “other beta-lactam antibacterials” (i.e., ATC code J01D).

The lowest and highest decreases in average antibiotics amount per prescription among the diagnosis of “acute upper respiratory infections” were 3.39% in July and 14.69% in September, respectively (Fig. 2A, Supplementary Table 4). The decrease in average amount from 2015 to 2016 was 8.33%.

For “acute upper respiratory infections”, decreases in average amount from 2015 to 2016 were 18.43% for “other beta-lactam antibacterials” (i.e., J01D ATC code), 10.46% for “sulfonamides and trimethoprim” (i.e., J01E ATC code), 7.89% for “macrolides, lincosamides and streptogramins” (i.e., J01F ATC code), 7.26% for “quinolone antibacterials” (i.e., J01M ATC code), and 0.93% for “beta-lactam antibacterials, penicilins” (i.e., J01C ATC code).

The highest decrease in average amount of “beta-lactam antibacterials, penicilins” per prescription among the diagnosis of “acute upper respiratory infections” was 8.57% in December, however there was a 5.53% increase in July (Fig. 2B, Supplementary Table 5). The decrease in average amount from 2015 to 2016 was 0.93%.

The lowest and highest decreases in average amount of “other beta-lactam antibacterials” per prescription among the diagnosis of “acute upper respiratory infections” were 11.57% in January and 24.85% in December, respectively (Fig. 2C, Supplementary Table 6). The decrease in average amount from 2015 to 2016 was 18.43%.

### Effect of ASP on antibiotics prescribed for acute upper respiratory infections in Denizli province

**Figure 3:**
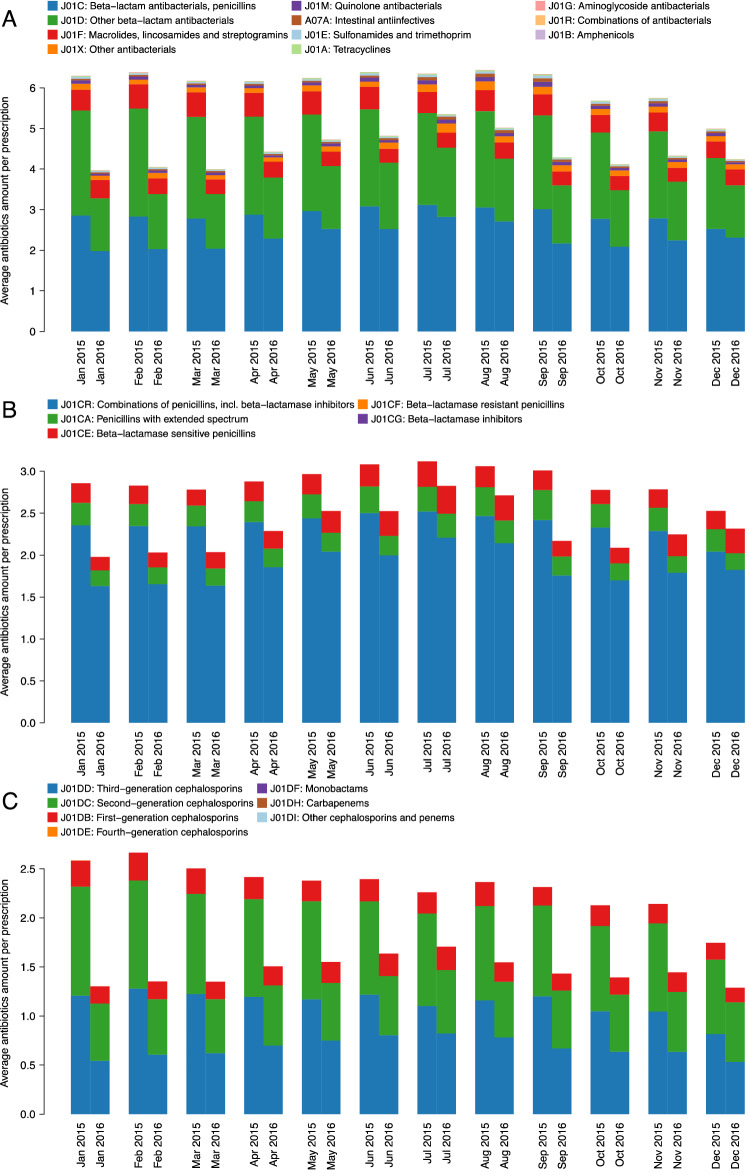
Monthly average antibiotics amounts per prescription for Denizli, where the highest decrease was observed, among the diagnosis of “acute upper respiratory infections” in 2015 and 2016. (**A**) Breakdown with respect to third level ATC codes, (**B**) Breakdown with respect to “beta-lactam antibacterials, penicillins” (i.e., ATC code J01C), and (**C**) Breakdown with respect to “other beta-lactam antibacterials” (i.e., ATC code J01D).

In Denizli province, the lowest and highest decreases in average antibiotics amount per prescription among the diagnosis of “acute upper respiratory infections” were 15.07% in December and 36.95% in January, respectively (Fig. 3A, Supplementary Table 7). The decrease in average amount from 2015 to 2016 was 28.63%.

The lowest and highest decreases in average amount of “beta-lactam antibacterials, penicilins” antibiotics per prescription among the diagnosis of “acute upper respiratory infections” were 8.38% in December and 30.68% in January, respectively. The decrease in average amount from 2015 to 2016 was 21.67% (Fig. 3B, Supplementary Table 8).

The lowest and highest decreases in average amount of “other beta-lactam antibacterials” per prescription among the diagnosis of “acute upper respiratory infections” were 24.53% in July and 49.63% in January, respectively (Fig. 3C, Supplementary Table 9). The decrease in average amount from 2015 to 2016 was 39.12%.

## Discussion

The selection of the outcome measure for antibiotics consumption is an important decision in ASP studies. In 2001, the European Surveillance of Antimicrobial Consumption (ESAC) project adopted the most widely recommended measure DDD^17^. More recently, measures other than DDD have been proposed to measure outpatient antibiotics consumption, e.g., the number of packages, the number of prescriptions, and the number of treated individuals^18^. DDD may not correspond the actual dose especially in paediatric patients, but it is the most widely-used measurement internationally^17^. Percentage of prescriptions with antibiotics was also suggested by World Health Organization especially for primary care centres. This percentage indicator does not consider the total antibiotics amount, and prescriptions with at least one dose of antibiotics were considered equally^17^. However, in our study, we included the total antibiotics amount, which is defined as number of boxes times number of items per box, in our calculations. The antibiotics amount that was used in our study accounts the number of items per box, as an additional advantage compared to using only number of boxes.

There are some limitations in our study. We could not have integrated the laboratory results, because the study was performed at FHUs, where diagnoses were usually done by clinical findings. We calculated the amount of the antibiotics by multiplying number of boxes and number of items per box. We could not calculate the DID values, however this limitation did not affect the outcome of our study, because we performed a pre- and post-intervention comparison using the same definition for the antibiotics amount^17^. The amount of the medicines in suspension form that would be usually used for paediatric patients were underestimated in our study since their amount will be calculated as one.

However, our study has several strong points. Firstly, by the advantage of using the data obtained from a nationwide healthcare system with universal coverage, we reached up to the highest number of participants in the literature. The big data was obtained from an actively working healthcare system, not from sales data, so that it included multifaceted information, which gave us an opportunity to perform further analyses.

## Conclusions

The increase in antibiotics consumption raises serious concerns for public health^16^. Turkey is a country with around 80 million inhabitants as of 2017, located between Europe and Asia. The proportion of the health expenditures in gross national product was reported as 6.1% in 2010^15^. Everybody is covered for access to the healthcare and has a family physician, and referral is not mandatory for the secondary and tertiary centres.

We presented a monitoring software for a nationwide ASP by collecting high-quality big data from healthcare system including at least one prescription for more than 50 million patients, which makes around 60% of the population in Turkey. In this respect, this study included by far the largest proportion (60%) of the population of a country with around 266 million prescriptions in primary care. After overall analysis, we focused on the most common diagnosis chapter “diseases of the respiratory system” and the most common diagnosis block “acute upper respiratory infections” (Table 1), in parallel with the rest of the world^11^.

Antibiotics consumption is significantly different between the regions in Turkey. In 79 out of 81 provinces, a decrease in average antibiotics amount in prescriptions from 2015 to 2016 was observed. The highest decrease was observed in Denizli province with 26.03%.

The most significant decrease was observed in “other beta-lactam antibacterials” category, particularly in “second-generation cephalosporins” (i.e., J01DC ATC code) and “third-generation cephalosporins” (i.e., J01DD ATC code) (Fig. 2). Turkey was previously reported as having the highest rate of consumption of “second-generation cephalosporins” and “third-generation cephalosporins” in 2011^12^.

This monitoring software supports decision makers for detecting strong and weak sides of ASPs. One important remaining question is the sustainability of such prescribing behaviour once the intervention is stopped. Some form of reinforcement or maintenance education is probably needed in order to sustain such a change in behaviour. Using the advantage of monitoring, we can modify the education and training in many different levels. Effective education programs for the rational use of antibiotics could be implemented at undergraduate level including medical, dental, pharmacy, and nursing schools, and at the medical residency programs. Internet-based learning for the physicians could be implemented for continuous education^11^. National dissemination of the information at public level is also important. The data obtained from this software could be used to enhance and sustain these strategies to decrease antibiotics consumption.

In conclusion, monitoring software that analyses big data collected from a nationwide healthcare system brought significant contributions in evaluating and improving ASPs.

## Supplementary Information

Supplementary Information.

## Data Availability

The results reported in this manuscript can be reproduced using the data in Supplementary Tables. The requests for raw data should be directed to the Turkish Medicines and Medical Devices Agency of Ministry of Health.
