# Supplementary material for: Assessment of quarter billion primary care prescriptions from a nationwide antimicrobial stewardship program
Source: Sci Rep. 2021 Jul 16;11:14621. doi: 10.1038/s41598-021-94308-z (PMC8285383; doi:10.1038/s41598-021-94308-z)
Supplement: Supplementary file 1 — Supplementary Information. [file 41598_2021_94308_MOESM1_ESM.docx]

**Supplementary Materials for “Assessment of Quarter Billion Primary Care Prescriptions from a Nationwide Antimicrobial Stewardship Program”**

Mehmet Gönen^1,2,*^, Mesil Aksoy^3^, Fatma İşli^3^, Umut Emre Gürpınar^3^, Pınar Göbel^3^, Hakkı Gürsöz^3^, Önder Ergönül^4^

^1^Department of Industrial Engineering, College of Engineering, Koç University, İstanbul, Turkey, ^2^School of Medicine, Koç University, İstanbul, Turkey, ^3^Turkish Medicines and Medical Devices Agency (TMMDA) of Ministry of Health, Ankara, Turkey, ^4^Department of Infectious Diseases and Clinical Microbiology, School of Medicine, Koç University, İstanbul, Turkey

* Corresponding author: [mehmetgonen@ku.edu.tr](mailto:mehmetgonen@ku.edu.tr)

**Supplementary Table 1. The antibiotics amount statistics for all prescriptions in provinces during 2015 and 2016.**

| **Province** | **Total antibiotics amount (2015)** | **Number of prescriptions (2015)** | **Average antibiotics amount per prescription (2015)** | **Total antibiotics amount (2016)** | **Number of prescriptions (2016)** | **Average antibiotics amount per prescription (2016)** | **Difference (2016 - 2015)** | **Difference (%)** |
| --- | --- | --- | --- | --- | --- | --- | --- | --- |
| DENİZLİ | 7711691 | 2553278 | 3.0203 | 5811942 | 2601572 | 2.2340 | -0.7863 | -26.0337 |
| ARDAHAN | 323007 | 129406 | 2.4961 | 223190 | 117246 | 1.9036 | -0.5925 | -23.7361 |
| KARS | 709028 | 265597 | 2.6696 | 552192 | 262582 | 2.1029 | -0.5666 | -21.2256 |
| BATMAN | 1127550 | 353421 | 3.1904 | 1091807 | 408877 | 2.6703 | -0.5201 | -16.3030 |
| ELAZIĞ | 2461714 | 825771 | 2.9811 | 2027903 | 803501 | 2.5238 | -0.4573 | -15.3391 |
| MUĞLA | 6488765 | 2085394 | 3.1115 | 5370536 | 2035662 | 2.6382 | -0.4733 | -15.2113 |
| DİYARBAKIR | 7122876 | 1722990 | 4.1340 | 6592597 | 1871731 | 3.5222 | -0.6118 | -14.7998 |
| GAZİANTEP | 11647106 | 2940526 | 3.9609 | 10703231 | 3142285 | 3.4062 | -0.5547 | -14.0044 |
| KONYA | 12904678 | 4114034 | 3.1367 | 10772955 | 3971619 | 2.7125 | -0.4243 | -13.5255 |
| IĞDIR | 757143 | 260603 | 2.9054 | 634323 | 248321 | 2.5544 | -0.3509 | -12.0778 |
| EDİRNE | 2383426 | 1035781 | 2.3011 | 2189960 | 1081931 | 2.0241 | -0.2770 | -12.0364 |
| SİVAS | 1783594 | 744751 | 2.3949 | 1623281 | 769428 | 2.1097 | -0.2852 | -11.9071 |
| BAYBURT | 272356 | 117000 | 2.3278 | 231549 | 112914 | 2.0507 | -0.2772 | -11.9065 |
| UŞAK | 3061242 | 878172 | 3.4859 | 2876924 | 933101 | 3.0832 | -0.4027 | -11.5533 |
| KIRKLARELİ | 2352693 | 862903 | 2.7265 | 2135796 | 882029 | 2.4215 | -0.3050 | -11.1876 |
| HATAY | 11312863 | 2848838 | 3.9710 | 10550278 | 2988039 | 3.5308 | -0.4402 | -11.0854 |
| NEVŞEHİR | 1630781 | 580428 | 2.8096 | 1351354 | 540012 | 2.5025 | -0.3072 | -10.9327 |
| KARAMAN | 1618730 | 490158 | 3.3025 | 1277092 | 433542 | 2.9457 | -0.3567 | -10.8025 |
| ŞANLIURFA | 6133049 | 1509524 | 4.0629 | 6197315 | 1708538 | 3.6273 | -0.4356 | -10.7224 |
| RİZE | 1467811 | 699541 | 2.0982 | 1244240 | 660989 | 1.8824 | -0.2159 | -10.2875 |
| ZONGULDAK | 2631071 | 1088709 | 2.4167 | 2264104 | 1036504 | 2.1844 | -0.2323 | -9.6133 |
| ERZİNCAN | 617039 | 280093 | 2.2030 | 530905 | 266186 | 1.9945 | -0.2085 | -9.4640 |
| AKSARAY | 2155701 | 655231 | 3.2900 | 1934302 | 647209 | 2.9887 | -0.3013 | -9.1582 |
| BİTLİS | 310324 | 132598 | 2.3403 | 143430 | 67427 | 2.1272 | -0.2131 | -9.1076 |
| AMASYA | 2030898 | 772814 | 2.6279 | 1860890 | 778734 | 2.3896 | -0.2383 | -9.0676 |
| ERZURUM | 1813612 | 850940 | 2.1313 | 1618388 | 833812 | 1.9410 | -0.1904 | -8.9313 |
| MANİSA | 8620604 | 3099360 | 2.7814 | 7378802 | 2912775 | 2.5333 | -0.2482 | -8.9221 |
| ESKİŞEHİR | 5031085 | 1585756 | 3.1727 | 4843305 | 1674730 | 2.8920 | -0.2807 | -8.8468 |
| BURDUR | 1405026 | 491886 | 2.8564 | 1184534 | 454827 | 2.6044 | -0.2520 | -8.8238 |
| AĞRI | 663240 | 254970 | 2.6012 | 538346 | 226981 | 2.3718 | -0.2295 | -8.8219 |
| SAKARYA | 5496762 | 1998804 | 2.7500 | 5073066 | 2021164 | 2.5100 | -0.2401 | -8.7291 |
| BALIKESİR | 7069716 | 2707897 | 2.6108 | 6819127 | 2859179 | 2.3850 | -0.2258 | -8.6481 |
| BOLU | 1426386 | 574608 | 2.4824 | 1357643 | 598618 | 2.2680 | -0.2144 | -8.6370 |
| KÜTAHYA | 4673288 | 1350930 | 3.4593 | 4357447 | 1377232 | 3.1639 | -0.2954 | -8.5391 |
| MERSİN | 16766959 | 3972151 | 4.2211 | 15934209 | 4125596 | 3.8623 | -0.3588 | -8.5012 |
| TUNCELİ | 185938 | 65792 | 2.8261 | 204577 | 79083 | 2.5869 | -0.2393 | -8.4668 |
| YOZGAT | 1188942 | 526007 | 2.2603 | 1139813 | 550859 | 2.0692 | -0.1912 | -8.4572 |
| MUŞ | 421166 | 190971 | 2.2054 | 378711 | 187474 | 2.0201 | -0.1853 | -8.4031 |
| YALOVA | 1202197 | 550758 | 2.1828 | 1124579 | 562096 | 2.0007 | -0.1821 | -8.3432 |
| TRABZON | 4444362 | 1749532 | 2.5403 | 4045339 | 1735736 | 2.3306 | -0.2097 | -8.2547 |
| ISPARTA | 2142416 | 810609 | 2.6430 | 1868672 | 770495 | 2.4253 | -0.2177 | -8.2363 |
| ADANA | 25035666 | 5612758 | 4.4605 | 23480001 | 5729240 | 4.0983 | -0.3622 | -8.1206 |
| ANKARA | 21916406 | 7585092 | 2.8894 | 21965662 | 8255708 | 2.6607 | -0.2287 | -7.9166 |
| ADIYAMAN | 2108546 | 666693 | 3.1627 | 2030776 | 696220 | 2.9169 | -0.2458 | -7.7729 |
| KIRŞEHİR | 805111 | 279099 | 2.8847 | 665588 | 249622 | 2.6664 | -0.2183 | -7.5674 |
| KİLİS | 931088 | 235608 | 3.9519 | 786150 | 215044 | 3.6558 | -0.2961 | -7.4924 |
| ÇORUM | 2337604 | 941384 | 2.4832 | 2204792 | 958858 | 2.2994 | -0.1838 | -7.4004 |
| KAYSERİ | 5916960 | 1929159 | 3.0671 | 6542740 | 2302357 | 2.8418 | -0.2254 | -7.3477 |
| TOKAT | 2028623 | 833744 | 2.4331 | 1932364 | 853896 | 2.2630 | -0.1702 | -6.9931 |
| BURSA | 17464514 | 5869620 | 2.9754 | 17220924 | 6218266 | 2.7694 | -0.2060 | -6.9234 |
| ÇANAKKALE | 3402991 | 1260793 | 2.6991 | 3156911 | 1255055 | 2.5154 | -0.1837 | -6.8072 |
| KARABÜK | 1284024 | 511941 | 2.5081 | 1235673 | 527936 | 2.3406 | -0.1676 | -6.6812 |
| ÇANKIRI | 946647 | 303365 | 3.1205 | 962911 | 330014 | 2.9178 | -0.2027 | -6.4958 |
| TEKİRDAĞ | 5950613 | 2138433 | 2.7827 | 5606632 | 2152372 | 2.6049 | -0.1778 | -6.3908 |
| BİNGÖL | 459496 | 175565 | 2.6172 | 353773 | 143902 | 2.4584 | -0.1588 | -6.0679 |
| KOCAELİ | 9010025 | 3282474 | 2.7449 | 8979895 | 3474969 | 2.5842 | -0.1607 | -5.8554 |
| KIRIKKALE | 1908684 | 566398 | 3.3699 | 1548631 | 488130 | 3.1726 | -0.1973 | -5.8544 |
| SAMSUN | 5190438 | 2012209 | 2.5795 | 6329438 | 2603816 | 2.4308 | -0.1486 | -5.7625 |
| ARTVİN | 431049 | 222095 | 1.9408 | 437605 | 239056 | 1.8306 | -0.1103 | -5.6820 |
| ORDU | 3166719 | 1223639 | 2.5880 | 3475695 | 1423250 | 2.4421 | -0.1459 | -5.6365 |
| MALATYA | 3335982 | 1096818 | 3.0415 | 3265390 | 1137460 | 2.8708 | -0.1707 | -5.6135 |
| İSTANBUL | 59625355 | 20469773 | 2.9128 | 61370396 | 22295384 | 2.7526 | -0.1602 | -5.5013 |
| DÜZCE | 2115111 | 753588 | 2.8067 | 1924950 | 725697 | 2.6526 | -0.1542 | -5.4928 |
| BİLECİK | 1105110 | 389827 | 2.8349 | 1136678 | 423946 | 2.6812 | -0.1537 | -5.4213 |
| SİNOP | 1132334 | 483491 | 2.3420 | 1060159 | 477395 | 2.2207 | -0.1213 | -5.1785 |
| ANTALYA | 11049059 | 3825332 | 2.8884 | 10400558 | 3790860 | 2.7436 | -0.1448 | -5.0133 |
| OSMANİYE | 3186664 | 733843 | 4.3424 | 3352315 | 810791 | 4.1346 | -0.2078 | -4.7856 |
| KAHRAMANMARAŞ | 5708265 | 1559068 | 3.6613 | 5499136 | 1577245 | 3.4865 | -0.1748 | -4.7738 |
| GİRESUN | 2005910 | 849251 | 2.3620 | 2018987 | 896922 | 2.2510 | -0.1110 | -4.6977 |
| KASTAMONU | 1425991 | 660868 | 2.1578 | 1479322 | 713607 | 2.0730 | -0.0847 | -3.9270 |
| GÜMÜŞHANE | 551708 | 189200 | 2.9160 | 503824 | 179816 | 2.8019 | -0.1141 | -3.9135 |
| MARDİN | 2970523 | 751243 | 3.9541 | 2802089 | 737405 | 3.7999 | -0.1542 | -3.9000 |
| AFYONKARAHİSAR | 4599288 | 1415088 | 3.2502 | 4584889 | 1467724 | 3.1238 | -0.1264 | -3.8881 |
| NİĞDE | 2119407 | 656808 | 3.2268 | 1877105 | 603060 | 3.1126 | -0.1142 | -3.5389 |
| AYDIN | 6381005 | 2381061 | 2.6799 | 5602387 | 2157883 | 2.5962 | -0.0837 | -3.1217 |
| İZMİR | 25412012 | 8719282 | 2.9145 | 24945233 | 8828794 | 2.8254 | -0.0890 | -3.0545 |
| BARTIN | 1366448 | 552450 | 2.4734 | 1225378 | 509163 | 2.4067 | -0.0668 | -2.6999 |
| ŞIRNAK | 854749 | 236693 | 3.6112 | 750283 | 210687 | 3.5611 | -0.0501 | -1.3870 |
| VAN | 1415040 | 500177 | 2.8291 | 1291641 | 456725 | 2.8280 | -0.0010 | -0.0364 |
| HAKKARİ | 123387 | 33645 | 3.6673 | 138186 | 36877 | 3.7472 | 0.0799 | 2.1785 |
| SİİRT | 372407 | 154193 | 2.4152 | 257729 | 103121 | 2.4993 | 0.0841 | 3.4816 |

**Supplementary Table 2. The antibiotics amount statistics for prescriptions with “diseases of the respiratory system” diagnosis in provinces during 2015 and 2016.**

| **Province** | **Total antibiotics amount (2015)** | **Number of prescriptions (2015)** | **Average antibiotics amount per prescription (2015)** | **Total antibiotics amount (2016)** | **Number of prescriptions (2016)** | **Average antibiotics amount per prescription (2016)** | **Difference (2016 - 2015)** | **Difference (%)** |
| --- | --- | --- | --- | --- | --- | --- | --- | --- |
| DENİZLİ | 5304008 | 942356 | 5.6285 | 3747422 | 925108 | 4.0508 | -1.5777 | -28.0301 |
| ARDAHAN | 179308 | 36038 | 4.9755 | 126435 | 33216 | 3.8064 | -1.1691 | -23.4966 |
| KARS | 429492 | 81313 | 5.2820 | 330456 | 75451 | 4.3797 | -0.9022 | -17.0811 |
| DİYARBAKIR | 5160436 | 890553 | 5.7946 | 4730968 | 968865 | 4.8830 | -0.9116 | -15.7325 |
| MUĞLA | 4394897 | 771406 | 5.6973 | 3499366 | 727419 | 4.8107 | -0.8866 | -15.5618 |
| GAZİANTEP | 8147777 | 1451860 | 5.6120 | 7388756 | 1555943 | 4.7487 | -0.8632 | -15.3819 |
| BATMAN | 737131 | 166802 | 4.4192 | 723499 | 193358 | 3.7418 | -0.6774 | -15.3295 |
| ELAZIĞ | 1275299 | 227069 | 5.6164 | 1020622 | 214093 | 4.7672 | -0.8492 | -15.1194 |
| EDİRNE | 1520906 | 309771 | 4.9098 | 1359304 | 320546 | 4.2406 | -0.6692 | -13.6297 |
| KONYA | 8798115 | 1573496 | 5.5914 | 7154769 | 1477219 | 4.8434 | -0.7480 | -13.3783 |
| KIRKLARELİ | 1611262 | 276104 | 5.8357 | 1428311 | 280241 | 5.0967 | -0.7390 | -12.6631 |
| SAKARYA | 3713476 | 739127 | 5.0241 | 3332437 | 753798 | 4.4209 | -0.6033 | -12.0075 |
| ZONGULDAK | 1824403 | 367669 | 4.9621 | 1538639 | 350371 | 4.3915 | -0.5706 | -11.4997 |
| HATAY | 7507312 | 1295684 | 5.7941 | 6926295 | 1349054 | 5.1342 | -0.6599 | -11.3893 |
| ŞANLIURFA | 4336685 | 781115 | 5.5519 | 4431625 | 896607 | 4.9427 | -0.6093 | -10.9738 |
| BİTLİS | 186401 | 39090 | 4.7685 | 84394 | 19835 | 4.2548 | -0.5137 | -10.7729 |
| YALOVA | 800563 | 184730 | 4.3337 | 720055 | 186172 | 3.8677 | -0.4660 | -10.7531 |
| BOLU | 962367 | 188604 | 5.1026 | 901884 | 196864 | 4.5813 | -0.5213 | -10.2169 |
| ADIYAMAN | 1428393 | 269134 | 5.3074 | 1359762 | 283936 | 4.7890 | -0.5184 | -9.7674 |
| ERZİNCAN | 397681 | 84901 | 4.6841 | 344654 | 81505 | 4.2286 | -0.4554 | -9.7230 |
| TUNCELİ | 114223 | 18794 | 6.0776 | 130673 | 23781 | 5.4948 | -0.5828 | -9.5890 |
| GÜMÜŞHANE | 356788 | 51692 | 6.9022 | 311296 | 49761 | 6.2558 | -0.6464 | -9.3647 |
| KAYSERİ | 3695153 | 673307 | 5.4881 | 3931626 | 788372 | 4.9870 | -0.5010 | -9.1298 |
| MERSİN | 11547181 | 1754045 | 6.5832 | 10729087 | 1792159 | 5.9867 | -0.5965 | -9.0608 |
| BİNGÖL | 253728 | 55364 | 4.5829 | 195402 | 46869 | 4.1691 | -0.4138 | -9.0291 |
| BALIKESİR | 4733510 | 873918 | 5.4164 | 4467742 | 905157 | 4.9359 | -0.4805 | -8.8721 |
| TRABZON | 2953123 | 574676 | 5.1388 | 2636776 | 562983 | 4.6836 | -0.4552 | -8.8578 |
| ERZURUM | 1228345 | 259609 | 4.7315 | 1096314 | 254187 | 4.3130 | -0.4185 | -8.8449 |
| UŞAK | 2056762 | 329295 | 6.2460 | 1880372 | 330036 | 5.6975 | -0.5485 | -8.7814 |
| MANİSA | 5791615 | 1061404 | 5.4566 | 4882494 | 980561 | 4.9793 | -0.4773 | -8.7468 |
| SİVAS | 1299132 | 246241 | 5.2759 | 1144691 | 237741 | 4.8149 | -0.4610 | -8.7377 |
| ADANA | 16689391 | 2576162 | 6.4784 | 15081743 | 2544522 | 5.9271 | -0.5513 | -8.5091 |
| ESKİŞEHİR | 3332523 | 555128 | 6.0032 | 3141605 | 570009 | 5.5115 | -0.4917 | -8.1900 |
| MARDİN | 1926883 | 341303 | 5.6457 | 1891813 | 364579 | 5.1890 | -0.4566 | -8.0882 |
| KARAMAN | 1174995 | 194674 | 6.0357 | 884383 | 159353 | 5.5498 | -0.4859 | -8.0499 |
| BAYBURT | 171781 | 35394 | 4.8534 | 148967 | 33367 | 4.4645 | -0.3889 | -8.0128 |
| KIRŞEHİR | 565779 | 94298 | 5.9999 | 462967 | 83842 | 5.5219 | -0.4780 | -7.9669 |
| ISPARTA | 1544024 | 292699 | 5.2751 | 1295219 | 266139 | 4.8667 | -0.4084 | -7.7425 |
| TOKAT | 1335433 | 250203 | 5.3374 | 1165830 | 236699 | 4.9254 | -0.4120 | -7.7197 |
| AMASYA | 1325760 | 249038 | 5.3235 | 1193721 | 242599 | 4.9206 | -0.4030 | -7.5697 |
| BURDUR | 1017170 | 185015 | 5.4978 | 845662 | 166325 | 5.0844 | -0.4134 | -7.5190 |
| ÇANAKKALE | 2300655 | 385841 | 5.9627 | 2108833 | 382109 | 5.5189 | -0.4438 | -7.4425 |
| BURSA | 12227096 | 2221453 | 5.5041 | 11907575 | 2336427 | 5.0965 | -0.4076 | -7.4056 |
| IĞDIR | 453286 | 95943 | 4.7245 | 390393 | 89214 | 4.3759 | -0.3486 | -7.3789 |
| MUŞ | 221487 | 49669 | 4.4593 | 198139 | 47941 | 4.1330 | -0.3263 | -7.3170 |
| TEKİRDAĞ | 4173575 | 806603 | 5.1743 | 3870649 | 807061 | 4.7960 | -0.3783 | -7.3108 |
| KOCAELİ | 6338263 | 1305898 | 4.8536 | 6199166 | 1377068 | 4.5017 | -0.3519 | -7.2494 |
| NEVŞEHİR | 1122647 | 205374 | 5.4664 | 947397 | 186853 | 5.0703 | -0.3961 | -7.2457 |
| YOZGAT | 748644 | 145700 | 5.1383 | 698083 | 146421 | 4.7676 | -0.3706 | -7.2128 |
| ÇORUM | 1653995 | 298477 | 5.5414 | 1532132 | 297146 | 5.1562 | -0.3853 | -6.9529 |
| ORDU | 1761677 | 342946 | 5.1369 | 1905733 | 398577 | 4.7813 | -0.3556 | -6.9215 |
| AKSARAY | 1487615 | 255426 | 5.8241 | 1326172 | 243926 | 5.4368 | -0.3873 | -6.6496 |
| ANKARA | 14280659 | 2545379 | 5.6104 | 13544547 | 2582871 | 5.2440 | -0.3664 | -6.5313 |
| RİZE | 1031188 | 223825 | 4.6071 | 863405 | 200355 | 4.3094 | -0.2977 | -6.4626 |
| KİLİS | 646877 | 107951 | 5.9923 | 557123 | 99277 | 5.6118 | -0.3805 | -6.3501 |
| SİNOP | 750816 | 151277 | 4.9632 | 672359 | 144651 | 4.6481 | -0.3150 | -6.3475 |
| ANTALYA | 7656679 | 1527287 | 5.0133 | 7083775 | 1507310 | 4.6996 | -0.3136 | -6.2562 |
| KÜTAHYA | 3050481 | 486746 | 6.2671 | 2832339 | 481790 | 5.8788 | -0.3883 | -6.1960 |
| KARABÜK | 880963 | 168350 | 5.2329 | 832853 | 169541 | 4.9124 | -0.3205 | -6.1252 |
| KAHRAMANMARAŞ | 3961195 | 668855 | 5.9224 | 3643475 | 655324 | 5.5598 | -0.3625 | -6.1216 |
| İSTANBUL | 41879137 | 8041038 | 5.2082 | 42053673 | 8600242 | 4.8898 | -0.3184 | -6.1125 |
| AĞRI | 362713 | 76991 | 4.7111 | 307771 | 69456 | 4.4312 | -0.2799 | -5.9422 |
| SAMSUN | 2831313 | 557349 | 5.0800 | 3321979 | 695129 | 4.7789 | -0.3010 | -5.9257 |
| DÜZCE | 1398336 | 283407 | 4.9340 | 1278897 | 274563 | 4.6579 | -0.2761 | -5.5955 |
| ÇANKIRI | 696770 | 103780 | 6.7139 | 674499 | 106021 | 6.3619 | -0.3520 | -5.2425 |
| BİLECİK | 751249 | 139045 | 5.4029 | 777081 | 151757 | 5.1206 | -0.2824 | -5.2260 |
| HAKKARİ | 79823 | 12007 | 6.6480 | 90564 | 14368 | 6.3032 | -0.3449 | -5.1875 |
| AYDIN | 4219311 | 772873 | 5.4593 | 3599898 | 695115 | 5.1789 | -0.2804 | -5.1363 |
| OSMANİYE | 2274759 | 330736 | 6.8779 | 2347055 | 358927 | 6.5391 | -0.3388 | -4.9257 |
| KIRIKKALE | 1334287 | 210450 | 6.3402 | 1042726 | 172775 | 6.0352 | -0.3050 | -4.8105 |
| İZMİR | 16389035 | 2984599 | 5.4912 | 15864163 | 3026679 | 5.2414 | -0.2498 | -4.5484 |
| NİĞDE | 1559563 | 280017 | 5.5695 | 1373569 | 258184 | 5.3201 | -0.2494 | -4.4782 |
| MALATYA | 2064801 | 353844 | 5.8353 | 1952356 | 350174 | 5.5754 | -0.2600 | -4.4548 |
| BARTIN | 847503 | 178531 | 4.7471 | 751999 | 165611 | 4.5408 | -0.2063 | -4.3466 |
| ŞIRNAK | 553777 | 100344 | 5.5188 | 504167 | 95189 | 5.2965 | -0.2223 | -4.0281 |
| SİİRT | 256409 | 60623 | 4.2296 | 171417 | 42193 | 4.0627 | -0.1669 | -3.9455 |
| GİRESUN | 1225831 | 255793 | 4.7923 | 1209872 | 260557 | 4.6434 | -0.1489 | -3.1065 |
| AFYONKARAHİSAR | 3135169 | 528641 | 5.9306 | 3127932 | 541556 | 5.7758 | -0.1548 | -2.6101 |
| ARTVİN | 246438 | 57027 | 4.3214 | 257411 | 60671 | 4.2427 | -0.0787 | -1.8210 |
| KASTAMONU | 880527 | 192651 | 4.5706 | 876521 | 194532 | 4.5058 | -0.0648 | -1.4175 |
| VAN | 845013 | 175665 | 4.8104 | 789184 | 161652 | 4.8820 | 0.0716 | 1.4890 |

**Supplementary Table 3. The antibiotics amount statistics for prescriptions with “acute upper respiratory infections” diagnosis in provinces during 2015 and 2016.**

| **Province** | **Total antibiotics amount (2015)** | **Number of prescriptions (2015)** | **Average antibiotics amount per prescription (2015)** | **Total antibiotics amount (2016)** | **Number of prescriptions (2016)** | **Average antibiotics amount per prescription (2016)** | **Difference (2016 - 2015)** | **Difference (%)** |
| --- | --- | --- | --- | --- | --- | --- | --- | --- |
| DENİZLİ | 4381218 | 727095 | 6.0256 | 3077262 | 715600 | 4.3003 | -1.7254 | -28.6342 |
| GAZİANTEP | 6434841 | 1096885 | 5.8665 | 5831039 | 1193687 | 4.8849 | -0.9816 | -16.7319 |
| ELAZIĞ | 1047115 | 183438 | 5.7083 | 832145 | 174050 | 4.7811 | -0.9272 | -16.2432 |
| ARDAHAN | 131363 | 23835 | 5.5113 | 96418 | 20836 | 4.6275 | -0.8839 | -16.0374 |
| BATMAN | 580201 | 127417 | 4.5536 | 578277 | 150941 | 3.8311 | -0.7224 | -15.8648 |
| DİYARBAKIR | 4083581 | 700819 | 5.8269 | 3810991 | 775568 | 4.9138 | -0.9131 | -15.6699 |
| KARS | 336145 | 54564 | 6.1606 | 254760 | 48916 | 5.2081 | -0.9525 | -15.4605 |
| MUĞLA | 3456345 | 555305 | 6.2242 | 2748477 | 518971 | 5.2960 | -0.9282 | -14.9129 |
| KIRKLARELİ | 1320858 | 204355 | 6.4635 | 1160725 | 206486 | 5.6213 | -0.8422 | -13.0303 |
| SAKARYA | 2979543 | 569930 | 5.2279 | 2691438 | 590217 | 4.5601 | -0.6678 | -12.7743 |
| KONYA | 7203150 | 1191094 | 6.0475 | 5795628 | 1097452 | 5.2810 | -0.7665 | -12.6750 |
| EDİRNE | 1160366 | 224548 | 5.1676 | 1050546 | 232750 | 4.5136 | -0.6539 | -12.6547 |
| ZONGULDAK | 1478675 | 267582 | 5.5261 | 1247904 | 257330 | 4.8494 | -0.6766 | -12.2444 |
| GÜMÜŞHANE | 229379 | 33288 | 6.8907 | 187034 | 30897 | 6.0535 | -0.8373 | -12.1507 |
| BİTLİS | 136861 | 26509 | 5.1628 | 61490 | 13503 | 4.5538 | -0.6090 | -11.7961 |
| ADIYAMAN | 1148068 | 203553 | 5.6401 | 1091883 | 219097 | 4.9836 | -0.6566 | -11.6412 |
| HATAY | 5796909 | 966834 | 5.9958 | 5309982 | 1000732 | 5.3061 | -0.6897 | -11.5026 |
| ŞANLIURFA | 3347266 | 597655 | 5.6007 | 3456630 | 691922 | 4.9957 | -0.6050 | -10.8018 |
| ERZİNCAN | 316687 | 60573 | 5.2282 | 276617 | 58938 | 4.6934 | -0.5348 | -10.2298 |
| BOLU | 751256 | 136117 | 5.5192 | 720847 | 144489 | 4.9889 | -0.5303 | -9.6074 |
| YALOVA | 605497 | 128296 | 4.7195 | 551317 | 128837 | 4.2792 | -0.4403 | -9.3304 |
| KAYSERİ | 2936862 | 505603 | 5.8086 | 3182014 | 602101 | 5.2849 | -0.5238 | -9.0173 |
| MERSİN | 9560927 | 1396439 | 6.8466 | 8843457 | 1418919 | 6.2325 | -0.6141 | -8.9696 |
| ADANA | 14070214 | 2088862 | 6.7358 | 12577225 | 2047609 | 6.1424 | -0.5934 | -8.8101 |
| KARAMAN | 982192 | 146218 | 6.7173 | 727731 | 118784 | 6.1265 | -0.5908 | -8.7953 |
| IĞDIR | 325421 | 63765 | 5.1034 | 280315 | 60194 | 4.6569 | -0.4466 | -8.7506 |
| BALIKESİR | 3777682 | 638727 | 5.9144 | 3556818 | 658967 | 5.3976 | -0.5168 | -8.7384 |
| BİNGÖL | 180528 | 36877 | 4.8954 | 144643 | 32243 | 4.4860 | -0.4094 | -8.3626 |
| MANİSA | 4760470 | 852853 | 5.5818 | 4027163 | 786832 | 5.1182 | -0.4636 | -8.3059 |
| KIRŞEHİR | 489026 | 74495 | 6.5645 | 403178 | 66903 | 6.0263 | -0.5382 | -8.1992 |
| ERZURUM | 1002452 | 195426 | 5.1296 | 915896 | 194468 | 4.7098 | -0.4198 | -8.1843 |
| TRABZON | 2323687 | 424640 | 5.4721 | 2098318 | 417520 | 5.0257 | -0.4465 | -8.1589 |
| BAYBURT | 143157 | 27353 | 5.2337 | 121547 | 25218 | 4.8199 | -0.4138 | -7.9071 |
| SİİRT | 211155 | 45392 | 4.6518 | 139445 | 32549 | 4.2842 | -0.3677 | -7.9035 |
| ESKİŞEHİR | 2839384 | 436362 | 6.5069 | 2700980 | 450634 | 5.9937 | -0.5132 | -7.8872 |
| TUNCELİ | 90862 | 13838 | 6.5661 | 105118 | 17358 | 6.0559 | -0.5102 | -7.7708 |
| UŞAK | 1624748 | 249701 | 6.5068 | 1511382 | 250583 | 6.0315 | -0.4753 | -7.3049 |
| KİLİS | 520557 | 81769 | 6.3662 | 449237 | 76102 | 5.9031 | -0.4631 | -7.2744 |
| TEKİRDAĞ | 3345853 | 608938 | 5.4946 | 3129926 | 614209 | 5.0959 | -0.3987 | -7.2564 |
| BURSA | 9537997 | 1577637 | 6.0457 | 9362296 | 1668628 | 5.6108 | -0.4350 | -7.1947 |
| KOCAELİ | 5178974 | 992329 | 5.2190 | 5114985 | 1056031 | 4.8436 | -0.3754 | -7.1932 |
| YOZGAT | 610138 | 103361 | 5.9030 | 547383 | 99784 | 5.4857 | -0.4173 | -7.0693 |
| TOKAT | 1004330 | 175256 | 5.7306 | 886847 | 166512 | 5.3260 | -0.4046 | -7.0607 |
| ÇORUM | 1407340 | 228380 | 6.1623 | 1297797 | 226433 | 5.7315 | -0.4308 | -6.9908 |
| DÜZCE | 1121248 | 214838 | 5.2190 | 1028382 | 211206 | 4.8691 | -0.3499 | -6.7052 |
| SİNOP | 560692 | 95385 | 5.8782 | 511785 | 93267 | 5.4873 | -0.3909 | -6.6498 |
| SİVAS | 1104868 | 186881 | 5.9121 | 972560 | 176213 | 5.5192 | -0.3929 | -6.6459 |
| ÇANAKKALE | 1562827 | 243011 | 6.4311 | 1441732 | 239908 | 6.0095 | -0.4216 | -6.5553 |
| ORDU | 1279144 | 228051 | 5.6090 | 1405884 | 267897 | 5.2479 | -0.3612 | -6.4391 |
| MARDİN | 1389315 | 248020 | 5.6016 | 1447051 | 275829 | 5.2462 | -0.3554 | -6.3452 |
| NEVŞEHİR | 901464 | 153045 | 5.8902 | 758093 | 137306 | 5.5212 | -0.3690 | -6.2646 |
| AKSARAY | 1093696 | 168979 | 6.4724 | 975438 | 160605 | 6.0735 | -0.3989 | -6.1624 |
| AMASYA | 1083261 | 188912 | 5.7342 | 989592 | 183830 | 5.3832 | -0.3510 | -6.1215 |
| BURDUR | 843954 | 141544 | 5.9625 | 706067 | 126116 | 5.5986 | -0.3639 | -6.1037 |
| İSTANBUL | 32594472 | 5885407 | 5.5382 | 33279919 | 6390955 | 5.2073 | -0.3308 | -5.9738 |
| KARABÜK | 700500 | 124493 | 5.6268 | 673417 | 127074 | 5.2994 | -0.3274 | -5.8188 |
| ISPARTA | 1287870 | 222011 | 5.8009 | 1100693 | 201357 | 5.4664 | -0.3346 | -5.7672 |
| KAHRAMANMARAŞ | 3148241 | 504546 | 6.2398 | 2979096 | 506481 | 5.8820 | -0.3578 | -5.7342 |
| ANTALYA | 6475527 | 1219407 | 5.3104 | 5984327 | 1195403 | 5.0061 | -0.3043 | -5.7298 |
| ANKARA | 11402106 | 1873957 | 6.0845 | 10902299 | 1900113 | 5.7377 | -0.3468 | -5.6997 |
| KÜTAHYA | 2570468 | 385964 | 6.6599 | 2420124 | 385251 | 6.2819 | -0.3779 | -5.6746 |
| AĞRI | 258425 | 49382 | 5.2332 | 219500 | 44416 | 4.9419 | -0.2913 | -5.5658 |
| RİZE | 844832 | 166934 | 5.0609 | 718675 | 150224 | 4.7840 | -0.2769 | -5.4704 |
| MUŞ | 154157 | 32791 | 4.7012 | 141858 | 31894 | 4.4478 | -0.2534 | -5.3902 |
| SAMSUN | 2162990 | 391534 | 5.5244 | 2534855 | 484804 | 5.2286 | -0.2958 | -5.3541 |
| ŞIRNAK | 431089 | 76265 | 5.6525 | 389985 | 72662 | 5.3671 | -0.2854 | -5.0491 |
| BARTIN | 635349 | 129904 | 4.8909 | 557370 | 119744 | 4.6547 | -0.2362 | -4.8300 |
| HAKKARİ | 67618 | 9934 | 6.8067 | 60732 | 9347 | 6.4975 | -0.3092 | -4.5431 |
| İZMİR | 13684915 | 2374540 | 5.7632 | 13340268 | 2423376 | 5.5048 | -0.2584 | -4.4829 |
| OSMANİYE | 1855870 | 252263 | 7.3569 | 1942513 | 275487 | 7.0512 | -0.3057 | -4.1551 |
| NİĞDE | 1335469 | 228473 | 5.8452 | 1201988 | 214428 | 5.6056 | -0.2396 | -4.0998 |
| ÇANKIRI | 548504 | 73011 | 7.5126 | 546891 | 75761 | 7.2186 | -0.2940 | -3.9132 |
| AYDIN | 3567978 | 622251 | 5.7340 | 3034904 | 549354 | 5.5245 | -0.2095 | -3.6535 |
| BİLECİK | 544006 | 87167 | 6.2410 | 572189 | 94803 | 6.0356 | -0.2054 | -3.2912 |
| KIRIKKALE | 1109461 | 163958 | 6.7667 | 889026 | 135786 | 6.5473 | -0.2195 | -3.2435 |
| MALATYA | 1692078 | 269272 | 6.2839 | 1617526 | 264146 | 6.1236 | -0.1603 | -2.5509 |
| ARTVİN | 207366 | 43474 | 4.7699 | 215887 | 46421 | 4.6506 | -0.1193 | -2.5001 |
| AFYONKARAHİSAR | 2583616 | 407812 | 6.3353 | 2584321 | 416070 | 6.2113 | -0.1240 | -1.9580 |
| GİRESUN | 897003 | 171310 | 5.2361 | 904220 | 175554 | 5.1507 | -0.0855 | -1.6324 |
| KASTAMONU | 641584 | 126591 | 5.0682 | 659415 | 129602 | 5.0880 | 0.0198 | 0.3914 |
| VAN | 692744 | 137390 | 5.0422 | 663222 | 127646 | 5.1958 | 0.1536 | 3.0467 |

**Supplementary Table 4. The monthly average antibiotics amounts for prescriptions with “acute upper respiratory infections” diagnosis for third level ATC codes during 2015 and 2016.**

| **Period** | **J01C** | **J01D** | **J01F** | **J01X** | **J01M** | **A07A** | **J01E** | **J01A** | **J01G** | **J01R** | **J01B** |
| --- | --- | --- | --- | --- | --- | --- | --- | --- | --- | --- | --- |
| Jan-15 | 2.7636 | 2.3600 | 0.4853 | 0.1175 | 0.0786 | 0.0353 | 0.0256 | 0.0151 | 0.0023 | 0.0000 | 0.0003 |
| Jan-16 | 2.7221 | 2.0869 | 0.5592 | 0.1123 | 0.0708 | 0.0367 | 0.0241 | 0.0120 | 0.0025 | 0.0000 | 0.0000 |
| Feb-15 | 2.8680 | 2.5712 | 0.5317 | 0.1186 | 0.0788 | 0.0364 | 0.0256 | 0.0164 | 0.0026 | 0.0000 | 0.0004 |
| Feb-16 | 2.7730 | 2.0669 | 0.5216 | 0.1293 | 0.0823 | 0.0386 | 0.0257 | 0.0163 | 0.0024 | 0.0000 | 0.0002 |
| Mar-15 | 2.7574 | 2.4203 | 0.5382 | 0.1055 | 0.0663 | 0.0332 | 0.0238 | 0.0133 | 0.0027 | 0.0000 | 0.0003 |
| Mar-16 | 2.8011 | 1.9502 | 0.4692 | 0.1157 | 0.0713 | 0.0366 | 0.0243 | 0.0158 | 0.0020 | 0.0000 | 0.0001 |
| Apr-15 | 2.8690 | 2.3202 | 0.5215 | 0.1175 | 0.0741 | 0.0363 | 0.0263 | 0.0155 | 0.0024 | 0.0000 | 0.0002 |
| Apr-16 | 2.9191 | 1.9364 | 0.4588 | 0.1204 | 0.0747 | 0.0401 | 0.0262 | 0.0166 | 0.0021 | 0.0000 | 0.0001 |
| May-15 | 2.9331 | 2.2001 | 0.4838 | 0.1346 | 0.0815 | 0.0397 | 0.0291 | 0.0178 | 0.0024 | 0.0000 | 0.0001 |
| May-16 | 2.9945 | 1.8748 | 0.4296 | 0.1335 | 0.0755 | 0.0485 | 0.0290 | 0.0174 | 0.0022 | 0.0000 | 0.0001 |
| Jun-15 | 3.0051 | 2.1849 | 0.4809 | 0.1561 | 0.0918 | 0.0515 | 0.0311 | 0.0197 | 0.0025 | 0.0000 | 0.0001 |
| Jun-16 | 3.0133 | 1.8660 | 0.4219 | 0.1516 | 0.0854 | 0.0546 | 0.0294 | 0.0195 | 0.0023 | 0.0000 | 0.0002 |
| Jul-15 | 3.0980 | 2.1035 | 0.4702 | 0.1829 | 0.1025 | 0.0675 | 0.0367 | 0.0241 | 0.0028 | 0.0000 | 0.0003 |
| Jul-16 | 3.2693 | 1.7941 | 0.3983 | 0.1883 | 0.0997 | 0.0730 | 0.0330 | 0.0240 | 0.0025 | 0.0000 | 0.0001 |
| Aug-15 | 3.0946 | 2.1357 | 0.4495 | 0.1990 | 0.1067 | 0.0908 | 0.0410 | 0.0232 | 0.0029 | 0.0000 | 0.0003 |
| Aug-16 | 3.1539 | 1.8297 | 0.4127 | 0.1809 | 0.0968 | 0.0802 | 0.0351 | 0.0227 | 0.0024 | 0.0000 | 0.0001 |
| Sep-15 | 3.1178 | 2.2498 | 0.4580 | 0.1943 | 0.1136 | 0.0854 | 0.0398 | 0.0230 | 0.0027 | 0.0000 | 0.0002 |
| Sep-16 | 2.8505 | 1.7546 | 0.3885 | 0.1635 | 0.0889 | 0.0659 | 0.0286 | 0.0190 | 0.0019 | 0.0000 | 0.0001 |
| Oct-15 | 2.7327 | 2.0751 | 0.4264 | 0.1406 | 0.0810 | 0.0555 | 0.0300 | 0.0159 | 0.0021 | 0.0000 | 0.0001 |
| Oct-16 | 2.5830 | 1.6176 | 0.3888 | 0.1274 | 0.0694 | 0.0487 | 0.0230 | 0.0146 | 0.0018 | 0.0001 | 0.0001 |
| Nov-15 | 2.7149 | 2.0463 | 0.4444 | 0.1424 | 0.0834 | 0.0441 | 0.0277 | 0.0175 | 0.0023 | 0.0000 | 0.0001 |
| Nov-16 | 2.6290 | 1.6102 | 0.3823 | 0.1284 | 0.0673 | 0.0451 | 0.0226 | 0.0149 | 0.0018 | 0.0005 | 0.0001 |
| Dec-15 | 2.6776 | 2.0166 | 0.4591 | 0.1245 | 0.0710 | 0.0400 | 0.0268 | 0.0145 | 0.0021 | 0.0000 | 0.0001 |
| Dec-16 | 2.7013 | 1.5156 | 0.4108 | 0.1186 | 0.0614 | 0.0423 | 0.0205 | 0.0127 | 0.0016 | 0.0033 | 0.0001 |

**Supplementary Table 5. The monthly average “beta-lactam antibacterials, penicilins” amounts for prescriptions with “acute upper respiratory infections” diagnosis during 2015 and 2016.**

| **Period** | **J01CR** | **J01CA** | **J01CE** | **J01CG** | **J01CF** |
| --- | --- | --- | --- | --- | --- |
| Jan-15 | 2.2869 | 0.2541 | 0.2226 | 0.0000 | 0.0000 |
| Jan-16 | 2.3207 | 0.2049 | 0.1964 | 0.0000 | 0.0000 |
| Feb-15 | 2.3854 | 0.2516 | 0.2310 | 0.0000 | 0.0000 |
| Feb-16 | 2.3303 | 0.2251 | 0.2176 | 0.0000 | 0.0000 |
| Mar-15 | 2.3292 | 0.2210 | 0.2072 | 0.0000 | 0.0000 |
| Mar-16 | 2.3604 | 0.2190 | 0.2217 | 0.0000 | 0.0000 |
| Apr-15 | 2.3974 | 0.2364 | 0.2353 | 0.0000 | 0.0000 |
| Apr-16 | 2.4385 | 0.2343 | 0.2462 | 0.0000 | 0.0000 |
| May-15 | 2.4245 | 0.2566 | 0.2519 | 0.0000 | 0.0000 |
| May-16 | 2.4726 | 0.2424 | 0.2795 | 0.0000 | 0.0000 |
| Jun-15 | 2.4724 | 0.2762 | 0.2565 | 0.0000 | 0.0000 |
| Jun-16 | 2.4731 | 0.2602 | 0.2800 | 0.0000 | 0.0000 |
| Jul-15 | 2.5480 | 0.2988 | 0.2513 | 0.0000 | 0.0000 |
| Jul-16 | 2.6845 | 0.3074 | 0.2775 | 0.0000 | 0.0000 |
| Aug-15 | 2.5636 | 0.2953 | 0.2356 | 0.0000 | 0.0000 |
| Aug-16 | 2.5989 | 0.2907 | 0.2642 | 0.0000 | 0.0000 |
| Sep-15 | 2.5795 | 0.3058 | 0.2325 | 0.0000 | 0.0000 |
| Sep-16 | 2.3600 | 0.2704 | 0.2201 | 0.0000 | 0.0000 |
| Oct-15 | 2.3021 | 0.2383 | 0.1922 | 0.0000 | 0.0000 |
| Oct-16 | 2.1478 | 0.2175 | 0.2177 | 0.0000 | 0.0000 |
| Nov-15 | 2.2672 | 0.2368 | 0.2109 | 0.0000 | 0.0000 |
| Nov-16 | 2.1574 | 0.2231 | 0.2484 | 0.0000 | 0.0000 |
| Dec-15 | 2.2513 | 0.2136 | 0.2127 | 0.0000 | 0.0000 |
| Dec-16 | 2.2148 | 0.2181 | 0.2685 | 0.0000 | 0.0000 |

**Supplementary Table 6. The monthly average “other beta-lactam antibacterials” amounts for prescriptions with “acute upper respiratory infections” diagnosis during 2015 and 2016.**

| **Period** | **J01DD** | **J01DC** | **J01DB** | **J01DE** | **J01DH** | **J01DF** | **J01DI** |
| --- | --- | --- | --- | --- | --- | --- | --- |
| Jan-15 | 1.0400 | 1.0295 | 0.2905 | 0.0000 | 0.0000 | 0.0000 | 0.0000 |
| Jan-16 | 0.9800 | 0.8466 | 0.2603 | 0.0000 | 0.0000 | 0.0000 | 0.0000 |
| Feb-15 | 1.1829 | 1.0750 | 0.3134 | 0.0000 | 0.0000 | 0.0000 | 0.0000 |
| Feb-16 | 1.0033 | 0.7888 | 0.2748 | 0.0000 | 0.0000 | 0.0000 | 0.0000 |
| Mar-15 | 1.1263 | 1.0133 | 0.2807 | 0.0000 | 0.0000 | 0.0000 | 0.0000 |
| Mar-16 | 0.9408 | 0.7423 | 0.2671 | 0.0000 | 0.0000 | 0.0000 | 0.0000 |
| Apr-15 | 1.0951 | 0.9749 | 0.2502 | 0.0000 | 0.0000 | 0.0000 | 0.0000 |
| Apr-16 | 0.9267 | 0.7300 | 0.2797 | 0.0000 | 0.0000 | 0.0000 | 0.0000 |
| May-15 | 1.0333 | 0.9221 | 0.2447 | 0.0000 | 0.0000 | 0.0000 | 0.0000 |
| May-16 | 0.9076 | 0.6927 | 0.2745 | 0.0000 | 0.0000 | 0.0000 | 0.0000 |
| Jun-15 | 1.0266 | 0.9025 | 0.2558 | 0.0000 | 0.0000 | 0.0000 | 0.0000 |
| Jun-16 | 0.9478 | 0.6510 | 0.2671 | 0.0000 | 0.0000 | 0.0000 | 0.0000 |
| Jul-15 | 0.9688 | 0.8788 | 0.2559 | 0.0000 | 0.0000 | 0.0000 | 0.0000 |
| Jul-16 | 0.8436 | 0.6743 | 0.2761 | 0.0000 | 0.0000 | 0.0000 | 0.0000 |
| Aug-15 | 1.0044 | 0.8757 | 0.2555 | 0.0000 | 0.0000 | 0.0000 | 0.0000 |
| Aug-16 | 0.8897 | 0.6721 | 0.2679 | 0.0000 | 0.0000 | 0.0000 | 0.0000 |
| Sep-15 | 1.0692 | 0.9078 | 0.2728 | 0.0000 | 0.0000 | 0.0000 | 0.0000 |
| Sep-16 | 0.8508 | 0.6526 | 0.2511 | 0.0000 | 0.0000 | 0.0000 | 0.0000 |
| Oct-15 | 0.9871 | 0.8398 | 0.2483 | 0.0000 | 0.0000 | 0.0000 | 0.0000 |
| Oct-16 | 0.7948 | 0.5948 | 0.2280 | 0.0000 | 0.0000 | 0.0000 | 0.0000 |
| Nov-15 | 0.9772 | 0.8262 | 0.2429 | 0.0000 | 0.0000 | 0.0000 | 0.0000 |
| Nov-16 | 0.7994 | 0.5785 | 0.2323 | 0.0000 | 0.0000 | 0.0000 | 0.0000 |
| Dec-15 | 0.9598 | 0.8151 | 0.2418 | 0.0000 | 0.0000 | 0.0000 | 0.0000 |
| Dec-16 | 0.6935 | 0.5997 | 0.2223 | 0.0000 | 0.0000 | 0.0000 | 0.0000 |

**Supplementary Table 7. The monthly average antibiotics amounts for prescriptions with “acute upper respiratory infections” diagnosis for third level ATC codes in Denizli province during 2015 and 2016.**

| **Period** | **J01C** | **J01D** | **J01F** | **J01X** | **J01M** | **A07A** | **J01E** | **J01A** | **J01G** | **J01R** | **J01B** |
| --- | --- | --- | --- | --- | --- | --- | --- | --- | --- | --- | --- |
| Jan-15 | 2.8558 | 2.5846 | 0.5149 | 0.1481 | 0.0823 | 0.0408 | 0.0479 | 0.0184 | 0.0066 | 0.0000 | 0.0002 |
| Jan-16 | 1.9796 | 1.3019 | 0.4513 | 0.1063 | 0.0489 | 0.0345 | 0.0302 | 0.0142 | 0.0049 | 0.0000 | 0.0000 |
| Feb-15 | 2.8298 | 2.6655 | 0.5950 | 0.1145 | 0.0750 | 0.0407 | 0.0369 | 0.0179 | 0.0074 | 0.0000 | 0.0002 |
| Feb-16 | 2.0310 | 1.3524 | 0.3909 | 0.1325 | 0.0582 | 0.0332 | 0.0332 | 0.0162 | 0.0043 | 0.0000 | 0.0000 |
| Mar-15 | 2.7812 | 2.5042 | 0.6071 | 0.1210 | 0.0594 | 0.0356 | 0.0373 | 0.0203 | 0.0061 | 0.0000 | 0.0000 |
| Mar-16 | 2.0353 | 1.3496 | 0.3591 | 0.1061 | 0.0628 | 0.0374 | 0.0262 | 0.0175 | 0.0026 | 0.0000 | 0.0000 |
| Apr-15 | 2.8775 | 2.4158 | 0.5836 | 0.1168 | 0.0680 | 0.0412 | 0.0421 | 0.0186 | 0.0042 | 0.0000 | 0.0000 |
| Apr-16 | 2.2867 | 1.5063 | 0.3869 | 0.1063 | 0.0516 | 0.0367 | 0.0305 | 0.0194 | 0.0023 | 0.0000 | 0.0000 |
| May-15 | 2.9670 | 2.3796 | 0.5705 | 0.1452 | 0.0762 | 0.0372 | 0.0397 | 0.0244 | 0.0042 | 0.0000 | 0.0000 |
| May-16 | 2.5254 | 1.5501 | 0.3548 | 0.1288 | 0.0715 | 0.0396 | 0.0309 | 0.0237 | 0.0043 | 0.0000 | 0.0000 |
| Jun-15 | 3.0820 | 2.3945 | 0.5517 | 0.1287 | 0.0946 | 0.0541 | 0.0515 | 0.0287 | 0.0043 | 0.0000 | 0.0000 |
| Jun-16 | 2.5245 | 1.6357 | 0.3413 | 0.1522 | 0.0601 | 0.0458 | 0.0364 | 0.0201 | 0.0028 | 0.0000 | 0.0000 |
| Jul-15 | 3.1189 | 2.2606 | 0.5186 | 0.1875 | 0.1022 | 0.0774 | 0.0545 | 0.0281 | 0.0034 | 0.0000 | 0.0000 |
| Jul-16 | 2.8255 | 1.7061 | 0.3655 | 0.2252 | 0.0962 | 0.0717 | 0.0326 | 0.0254 | 0.0057 | 0.0000 | 0.0000 |
| Aug-15 | 3.0593 | 2.3649 | 0.5201 | 0.2155 | 0.1040 | 0.0872 | 0.0511 | 0.0351 | 0.0051 | 0.0000 | 0.0000 |
| Aug-16 | 2.7127 | 1.5463 | 0.3978 | 0.1530 | 0.0778 | 0.0725 | 0.0311 | 0.0231 | 0.0042 | 0.0000 | 0.0000 |
| Sep-15 | 3.0100 | 2.3143 | 0.5182 | 0.1838 | 0.1239 | 0.0885 | 0.0648 | 0.0284 | 0.0057 | 0.0000 | 0.0005 |
| Sep-16 | 2.1691 | 1.4325 | 0.3444 | 0.1514 | 0.0771 | 0.0612 | 0.0308 | 0.0218 | 0.0022 | 0.0000 | 0.0000 |
| Oct-15 | 2.7763 | 2.1266 | 0.4311 | 0.1467 | 0.0823 | 0.0477 | 0.0488 | 0.0221 | 0.0030 | 0.0000 | 0.0000 |
| Oct-16 | 2.0890 | 1.3918 | 0.3514 | 0.1348 | 0.0589 | 0.0441 | 0.0264 | 0.0150 | 0.0028 | 0.0000 | 0.0005 |
| Nov-15 | 2.7850 | 2.1425 | 0.4666 | 0.1456 | 0.0823 | 0.0515 | 0.0491 | 0.0232 | 0.0041 | 0.0000 | 0.0000 |
| Nov-16 | 2.2464 | 1.4454 | 0.3384 | 0.1415 | 0.0616 | 0.0438 | 0.0282 | 0.0203 | 0.0035 | 0.0014 | 0.0000 |
| Dec-15 | 2.5267 | 1.7462 | 0.4112 | 0.1290 | 0.0712 | 0.0451 | 0.0373 | 0.0199 | 0.0046 | 0.0000 | 0.0000 |
| Dec-16 | 2.3148 | 1.2876 | 0.3942 | 0.1201 | 0.0483 | 0.0385 | 0.0189 | 0.0135 | 0.0022 | 0.0007 | 0.0000 |

**Supplementary Table 8. The monthly average “beta-lactam antibacterials, penicilins” amounts for prescriptions with “acute upper respiratory infections” diagnosis in Denizli province during 2015 and 2016.**

| **Period** | **J01CR** | **J01CA** | **J01CE** | **J01CF** | **J01CG** |
| --- | --- | --- | --- | --- | --- |
| Jan-15 | 2.3569 | 0.2659 | 0.2330 | 0.0000 | 0.0000 |
| Jan-16 | 1.6318 | 0.1881 | 0.1598 | 0.0000 | 0.0000 |
| Feb-15 | 2.3457 | 0.2647 | 0.2194 | 0.0000 | 0.0000 |
| Feb-16 | 1.6538 | 0.2008 | 0.1764 | 0.0000 | 0.0000 |
| Mar-15 | 2.3439 | 0.2473 | 0.1900 | 0.0000 | 0.0000 |
| Mar-16 | 1.6365 | 0.2049 | 0.1938 | 0.0000 | 0.0000 |
| Apr-15 | 2.3946 | 0.2492 | 0.2337 | 0.0000 | 0.0000 |
| Apr-16 | 1.8568 | 0.2212 | 0.2087 | 0.0000 | 0.0000 |
| May-15 | 2.4395 | 0.2876 | 0.2398 | 0.0000 | 0.0000 |
| May-16 | 2.0442 | 0.2226 | 0.2586 | 0.0000 | 0.0000 |
| Jun-15 | 2.5027 | 0.3160 | 0.2634 | 0.0000 | 0.0000 |
| Jun-16 | 1.9972 | 0.2334 | 0.2940 | 0.0000 | 0.0000 |
| Jul-15 | 2.5211 | 0.2939 | 0.3038 | 0.0000 | 0.0000 |
| Jul-16 | 2.2073 | 0.2860 | 0.3322 | 0.0000 | 0.0000 |
| Aug-15 | 2.4662 | 0.3444 | 0.2487 | 0.0000 | 0.0000 |
| Aug-16 | 2.1423 | 0.2720 | 0.2984 | 0.0000 | 0.0000 |
| Sep-15 | 2.4183 | 0.3578 | 0.2339 | 0.0000 | 0.0000 |
| Sep-16 | 1.7572 | 0.2263 | 0.1856 | 0.0000 | 0.0000 |
| Oct-15 | 2.3301 | 0.2793 | 0.1668 | 0.0000 | 0.0000 |
| Oct-16 | 1.6985 | 0.2037 | 0.1867 | 0.0000 | 0.0000 |
| Nov-15 | 2.2883 | 0.2789 | 0.2178 | 0.0000 | 0.0000 |
| Nov-16 | 1.7914 | 0.1963 | 0.2587 | 0.0000 | 0.0000 |
| Dec-15 | 2.0421 | 0.2662 | 0.2183 | 0.0000 | 0.0000 |
| Dec-16 | 1.8252 | 0.1975 | 0.2921 | 0.0000 | 0.0000 |

**Supplementary Table 9. The monthly average “other beta-lactam antibacterials” amounts for prescriptions with “acute upper respiratory infections” diagnosis in Denizli province during 2015 and 2016.**

| **Period** | **J01DD** | **J01DC** | **J01DB** | **J01DE** | **J01DF** | **J01DH** | **J01DI** |
| --- | --- | --- | --- | --- | --- | --- | --- |
| Jan-15 | 1.2065 | 1.1125 | 0.2655 | 0.0001 | 0.0000 | 0.0000 | 0.0000 |
| Jan-16 | 0.5427 | 0.5858 | 0.1734 | 0.0000 | 0.0000 | 0.0000 | 0.0000 |
| Feb-15 | 1.2795 | 1.1028 | 0.2832 | 0.0000 | 0.0000 | 0.0000 | 0.0000 |
| Feb-16 | 0.6054 | 0.5675 | 0.1795 | 0.0000 | 0.0000 | 0.0000 | 0.0000 |
| Mar-15 | 1.2241 | 1.0198 | 0.2603 | 0.0000 | 0.0000 | 0.0000 | 0.0000 |
| Mar-16 | 0.6215 | 0.5511 | 0.1770 | 0.0000 | 0.0000 | 0.0000 | 0.0000 |
| Apr-15 | 1.1949 | 0.9974 | 0.2235 | 0.0000 | 0.0000 | 0.0000 | 0.0000 |
| Apr-16 | 0.6982 | 0.6138 | 0.1943 | 0.0000 | 0.0000 | 0.0000 | 0.0000 |
| May-15 | 1.1708 | 1.0007 | 0.2081 | 0.0000 | 0.0000 | 0.0000 | 0.0000 |
| May-16 | 0.7497 | 0.5874 | 0.2130 | 0.0000 | 0.0000 | 0.0000 | 0.0000 |
| Jun-15 | 1.2183 | 0.9509 | 0.2253 | 0.0000 | 0.0000 | 0.0000 | 0.0000 |
| Jun-16 | 0.8046 | 0.6043 | 0.2268 | 0.0000 | 0.0000 | 0.0000 | 0.0000 |
| Jul-15 | 1.1021 | 0.9427 | 0.2157 | 0.0000 | 0.0000 | 0.0000 | 0.0000 |
| Jul-16 | 0.8226 | 0.6463 | 0.2372 | 0.0000 | 0.0000 | 0.0000 | 0.0000 |
| Aug-15 | 1.1595 | 0.9625 | 0.2429 | 0.0000 | 0.0000 | 0.0000 | 0.0000 |
| Aug-16 | 0.7815 | 0.5682 | 0.1964 | 0.0002 | 0.0000 | 0.0000 | 0.0000 |
| Sep-15 | 1.2028 | 0.9230 | 0.1886 | 0.0000 | 0.0000 | 0.0000 | 0.0000 |
| Sep-16 | 0.6702 | 0.5907 | 0.1716 | 0.0000 | 0.0000 | 0.0000 | 0.0000 |
| Oct-15 | 1.0492 | 0.8686 | 0.2088 | 0.0000 | 0.0000 | 0.0000 | 0.0000 |
| Oct-16 | 0.6362 | 0.5816 | 0.1740 | 0.0000 | 0.0000 | 0.0000 | 0.0000 |
| Nov-15 | 1.0454 | 0.8991 | 0.1980 | 0.0000 | 0.0000 | 0.0000 | 0.0000 |
| Nov-16 | 0.6346 | 0.6122 | 0.1986 | 0.0000 | 0.0000 | 0.0000 | 0.0000 |
| Dec-15 | 0.8161 | 0.7580 | 0.1722 | 0.0000 | 0.0000 | 0.0000 | 0.0000 |
| Dec-16 | 0.5328 | 0.6072 | 0.1476 | 0.0000 | 0.0000 | 0.0000 | 0.0000 |
